# A new species of the Southeast Asian genus *Opisthotropis* (Serpentes: Colubridae: Natricinae) from western Hunan, China

**DOI:** 10.24272/j.issn.2095-8137.2017.068

**Published:** 2017-09-18

**Authors:** Jin-Long Ren, Kai Wang, Ke Jiang, Peng Guo, Jia-Tang Li

**Affiliations:** ^1^CAS Key Laboratory of Mountain Ecological Restoration and Bioresource Utilization & Ecological Restoration and Biodiversity Conservation Key Laboratory of Sichuan Province, Chengdu Institute of Biology, Chinese Academy of Sciences, Chengdu Sichuan 610041, China; ^2^Southeast Asia Biodiversity Research Institute, Chinese Academy of Sciences, Yezin Nay Pyi Taw 05282, Myanmar; ^3^University of Chinese Academy of Sciences, Beijing 100049, China; ^4^Sam Noble Oklahoma Museum of Natural History & Department of Biology, University of Oklahoma, Norman Oklahoma 73019, USA; ^5^College of Life Sciences and Food Engineering, Yibin University, Yibin Sichuan 644007, China

**Keywords:** Distribution, Natricinae, Natural history, Taxonomy, *Opisthotropis*, *Opisthotropis zhaoermii* sp. nov.

## Abstract

A new species of natricine snake of the Southeast Asian genus *Opisthotropis* Günther, 1872 is described from western Hunan Province of China based on both mitochondrial DNA and morphological data. The new species is morphologically most similar and genetically most closely related to *O. cheni* Zhao, 1999 and *O. latouchii* (Boulenger, 1899), but possesses considerable genetic divergence (*p*-distance 5.1%-16.7%) and can be differentiated from all other congeners by a combination of the following morphological characters: (1) body size large (total length 514-586 mm) and strongly built; (2) dorsal scale rows 17 throughout, feebly keeled anteriorly and moderately keeled posteriorly; (3) ventral scales 147-152, subcaudal scales 54-62; (4) preocular absent, loreal elongated and touching orbit; (5) supralabials 8-9, fifth and sixth entering obit; (6) anterior temporals short, length 1.74-2.04 times longer than width; (7) maxillary teeth subequal, 28-30; (8) dorsal surface of head with distinct irregular yellow stripes and markings edged with ochre; (9) body with clear black and yellow longitudinal streaks, partly fused to several lighter patches or thicker stripes anteriorly; and (10) venter pale yellow, with asymmetric blackish speckles along outer margin. We present an updated diagnostic key to all members of the genus *Opisthotropis*, and recommendations on the ecological study for the group are provided.

## INTRODUCTION

The snakes of the family Colubridae represent the most specious group of extant Serpentes (Figueroa et al., 2016; Pyron et al., 2013). Within this diverse group, keelback snakes of the subfamily Natricinae exhibit adaptations to aquatic habitats to different degrees, and include aquatic specialists of the genus *Opisthotropis* Günther, 1872 (Guo et al., 2014; Zhao et al., 1998). The generic nomenclature of *Opisthotropis* means "keeled backwards", based on its distinct morphological adaptations for an aquatic lifestyle (Günther, 1872; Ziegler et al., 2008). The natricine genus *Opisthotropis* is distributed across southern China and Southeast Asia, and inhabits flowing streams or waterfalls in low to moderate altitude forested areas (Pope, 1935; Zhao, 2006). Currently, the genus *Opisthotropis* includes 22 species, with 12 currently recorded in China: *O*. *andersonii* (Boulenger, 1888), *O*. *balteata* (Cope, 1895), *O*. *latouchii* (Boulenger, 1899), *O*. *lateralis* Boulenger, 1903, *O*. *maxwelli* Boulenger, 1914, *O*. *kuatunensis* Pope, 1928, *O*. *jacobi* Angel and Bourret, 1933, *O*. *guangxiensis* Zhao, Jiang and Huang, 1978, *O*. *cheni* Zhao, 1999, *O*. *maculosa* Stuart and Chuaynkern, 2007, *O*. *laui* Yang, Sung and Chan, 2013, and *O*.*shenzhenensis* Wang, Guo, Liu, Lyu, Wang, Luo, Sun, and Zhang, 2017 (Cai et al., 2015; Murphy et al., 2008; Wang et al., 2017).

Despite the remarkable ecological adaptations and rich diversity of *Opisthotropis*, little attention has been given to its taxonomy until recently (Chuaynkern et al., 2014; David et al., 2011; Iskandar & Kamsi, 2009; Murphy et al., 2008; Wang et al., 2017). This is particularly true for congeners in southern China. Prior to 2010, no modern taxonomic work on Chinese *Opisthotropis* had been conducted, and our understandings of the genus were based on study that dated back to the nineteenth century (Pope, 1935; Smith, 1943; Taylor, 1965; Zhao & Adler, 1993; Zhao et al., 1998; Zheng, 1992). Although a few recent taxonomic works using genetic methods have revealed surprising endemism and cryptic diversity within the genus, these studies have focused on areas around the Fujian-Guangdong Coast Subregion of southern China, with many areas in southern China remaining under-surveyed in regard to *Opisthotropis* diversity (Wang et al., 2017; Yang et al., 2011, 2013). As *Opisthotropis* congeners are ecological specialists with narrow niches and limited dispersal abilities, it is possible that previous records of widespread congeners may represent overlooked distinct and cryptic species (Wang et al., 2017; Zhao et al., 1998; Zhao, 2006).

During field surveys of Hunan Province in 2017, three adult specimens of *Opisthotropis* were collected in western Hunan, China. Detailed morphological comparisons and phylogenetic analyses showed that the western Hunan population of *Opisthotropis* represents a distinct evolutionary lineage that can be diagnosed readily from closely related congeners. Therefore, we describe the Hunan population of *Opisthotropis* as a new species herein.

## MATERIALS AND METHODS

### Sampling

**Figure 1:**
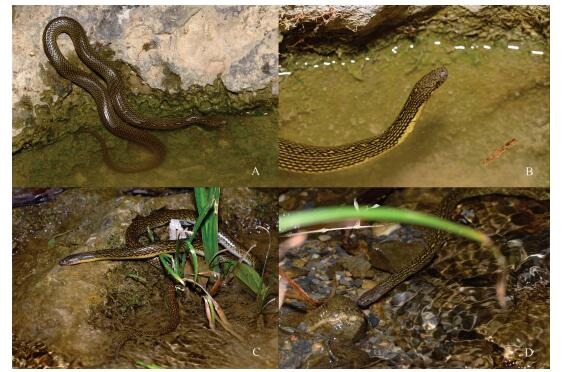
General and close-up views of *Opisthotropis zhaoermii* sp. nov. holotype CIB109999 (adult female) and paratype CIB109998 (adult male) in life (Photos by Jin-Long Ren)

**Figure 2:**
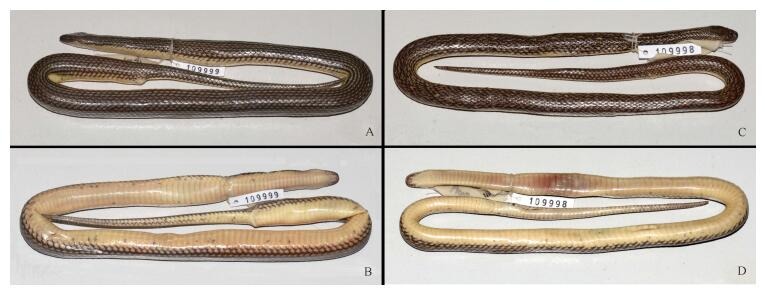
Dorsal and ventral views of *Opisthotropis zhaoermii* sp. nov. holotype CIB109999 (adult female) and CIB109998 (adult male) (Photos by Jin-Long Ren)

**Figure 3:**
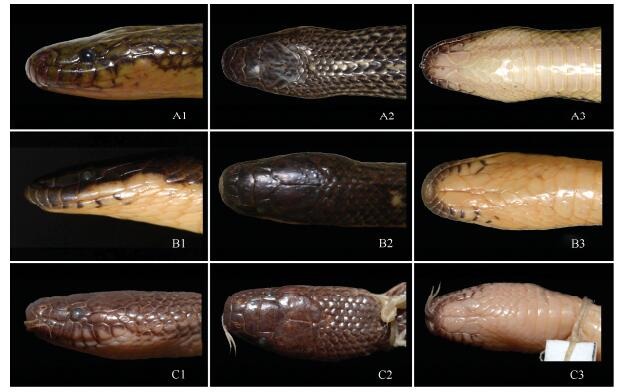
Lateral head (column 1), dorsal head (column 2), and ventral head (column 3) comparisons of *Opisthotropis zhaoermii* sp. nov., *O*. *cheni*, and *O*. *latouchii* (Photos by Jin-Long Ren)

**Figure 4:**
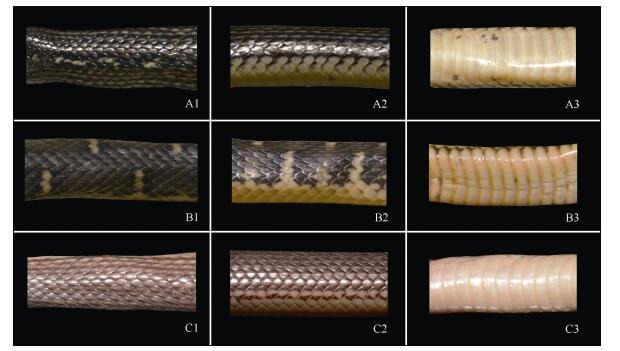
Anterior dorsal (column 1), dorsolateral (column 2), and ventral coloration (column 3) comparisons of *Opisthotropis zhaoermii* sp. nov., *O*. *cheni*, and *O*. *latouchii* (Photos by Jin-Long Ren)

**Figure 6:**
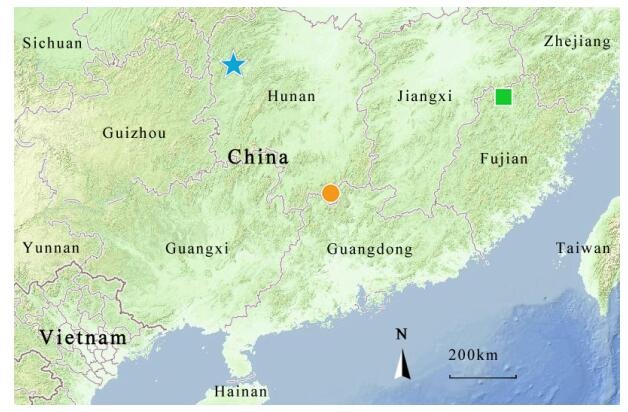
Type localities of species of the <italic>Opisthotropis latouchii</italic> group (created from MAP WORLD, <a href="http://www.tianditu.com">http://www.tianditu.com</a>)

Three adult *Opisthotropis* specimens (two females and one male) were collected from Guzhang, western Hunan, China on 22, 23, and 24 August 2017 (Figures 1, 2, 3A, 4A, 6). After each collection, the temperature and pH of the water were measured immediately using a liquid-in-glass thermometer and digital pH meter (calibrated), respectively. After euthanization, liver tissues were taken and preserved in 95% ethanol for DNA extraction, with all specimens preserved in 10% formalin in the field and transferred to 75% ethanol for permanent storage after fieldwork. All vouchered specimens and tissue samples were deposited in the Museum of Herpetology, Chengdu Institute of Biology (CIB), Chinese Academy of Sciences (CAS).

### Molecular analyses

#### DNA extraction, amplification, and sequencing

Genomic DNA was extracted from macerated liver tissue samples using a TIANamp Genomic DNA kit (Tiangen Biotech, China), according to the protocols of the manufacturer. Mitochondrial gene cytochrome *b* (cyt *b*) was targeted and amplified using primers L14919 (5'–AACCACCGTTGTTATT CAACT–3') and H16064 (5'–CTTTGGTTTACAAGAACAATGC TTTA–3') (Burbrink et al., 2000; Guo et al., 2012). Polymerase chain reactions (PCR) were performed at 25 μL volume, and amplified DNA was produced after initial denaturing for 7 min at 94 ℃, 41 cycles of denaturation for 40 s at 94 ℃, annealing for 30 s at 46 ℃, extension for 1 min at 72 ℃, and final extension for 8 min at 72 ℃. The PCR products were purified using commercial kits and sequenced in both directions by an ABI 3730xL sequencer (Applied Biosystems, Foster City, CA, USA). Sequence editing and management were performed in Geneious Pro 4.8.4 (Kearse et al., 2012). All new sequences were deposited in GenBank (Table 1).

**Table 1:** Samples and sequences used in molecular analysis

Genus	Species	Locality	Voucher No.	GenBank accession No.
*Opisthotropis*	*zhaoermii* **sp. nov**	Guzhang County, Hunan Province, China	CIB109998	MG012799*
	*zhaoermii* **sp. nov**	Guzhang County, Hunan Province, China	CIB109999	MG012800*
	*zhaoermii* **sp. nov**	Guzhang County, Hunan Province, China	CIB110000	MG012801*
	*cheni*	Nanling National Nature Reserve, Ruyuan County, Guangdong Province, China	YBU071040	GQ281779*
	*cheni*	Shimentai Nature Reserve, Yingde City, Guangdong Province, China	SYS r001422	KY594741*
	*latouchii*	Fujian Province, China	–	GQ281783*
	*latouchii*	Guadun Village, Wuyishan City, Fujian Province, China	SYS r000670	KY594742*
	*kuatunensis*	Qixiling Nature Reserve, Yongxin County, Jiangxi Province, China	SYS r000998	KY594745*
	*kuatunensis*	Wulong Village, Shanghang County, Fujian Province, China	SYS r001008	KY594746
	*kuatunensis*	Mt. Wutong, Shenzhen City, Guangdong Province, China	SYS r001081	KY594747
	*lateralis*	Guangxi Province, China	–	GQ281782*
	*lateralis*	Heishiding Nature Reserve, Fengkai County, Guangdong Province, China	SYS r000951	KY594743
	*lateralis*	Mt. Wutong, Shenzhen, Guangdong Province, China	SYS r001080	KY594744
	*laui*	Shangchuan Island, Taishan County, Guangdong Province, China	SYS r001161	KY594738*
	*laui*	Shangchuan Island, Taishan County, Guangdong Province, China	SYS r001170	KY594739
	*laui*	Shangchuan Island, Taishan County, Guangdong Province, China	SYS r001171	KY594740
	*maxwelli*	Nan'ao Island, Nan'ao County, Guangdong Province, China	SYS r000841	KY594736*
	*maxwelli*	Huboliao Nature Reserve, Nanjing County, Fujian Province, China	SYS r001053	KY594737
	*andersonii*	Mt. Wutong, Shenzhen City, Guangdong Province, China	SYS r001020	KY594732*
	*andersonii*	Mt. Wutong, Shenzhen City, Guangdong Province, China	SYS r001082	KY594733
	*andersonii*	Mt. Maofeng, Guangzhou City, Guangdong Province, China	SYS r001382	KY594734
*Opisthotropis*	*andersonii*	Mt. Maofeng, Guangzhou City, Guangdong Province, China	SYS r001383	KY594735
	*andersonii*	Tai Tam, HK Island, Hong Kong	SYS r001423	KY594730
	*andersonii*	Tai Mo Shan, Hong Kong	SYS r001424	KY594731
	*shenzhenensis*	Mt. Wutong, Shenzhen, Guangdong Province, China	SYS r001018	KY594727*
	*shenzhenensis*	Sanzhoutian, Shenzhen City, Guangdong Province, China	SYS r001021	KY594728
	*shenzhenensis*	Mt. Tiantou, Shenzhen City, Guangdong Province, China	SYS r001032	KY594729
	*maculosa*	Heishiding Nature Reserve, Fengkai County, Guangdong Province, China	SYS r000946	KY594748*
	*guangxiensis*	Guangxi Province, China	–	GQ281776*
*Natrix*	*natrix*	S Borup, Zealand, Denmark	MTD T 9269	HF680010
*Rhabdophis*	*leonardi*	Panzhihua City, Sichuan Province, China	SCUM090009	KF800933
*Hebius*	*johannis*	Yunnan Province, China	GP897	KJ685708
GenBank accession No. marked with * represents sequence used in the genetic distance analysis to represent respective species; "–" indicates missing voucher information.				

### Phylogenetic analyses

*Natrix natrix* (Linnaeus, 1758), *Rhabdophis leonardi* (Wall, 1923), and *Hebius johannis* (Boulenger, 1908) were selected as the outgroups for phylogenetic analyses (Figueroa et al., 2016). We downloaded all 26 available cyt *b* sequences of congeners and the above outgroups from GenBank (Table 1). The dataset was aligned using MEGA 6 with default parameters (Tamura et al., 2013), and adjusted manually. Possible saturation of substitution types was checked using DAMBE (Xia & Xie, 2001).

Bayesian inference (BI) and maximum likelihood (ML) analyses were conducted. The DNA substitution model was calculated by PartitionFinder 2 (Lanfear et al., 2017) using Akaike Information Criterion (AIC), which is a GTR+I+G model for the first and second codon positions and a TIM+G model for the third position. The BI analyses were conducted using MrBayes 3.1.2, as described in Ronquist & Huelsenbeck (2003). Two independent runs of four Markov Chains for 10 000 000 generations were summarized by the BI, and sampled every 100 generations. The first 25% were discarded as "burn-in". Bayesian posterior probability (BPP) was determined to test the confidence of tree topology, where nodes with BPP≥95% were considered strongly supported. Convergence and effective sample size (ESS) of the parameters were investigated in Tracer 1.6 (Rambaut & Drummond, 2013).

The ML analyses were performed in RAxML 8.2.10 (Stamatakis, 2014) under the most complex substitution model (GTRGAMMA) based on the AIC model assessment results. Partitions were unlinked and bootstrap proportions (BSP) were investigated with 1 000 bootstrap replicates using the fast bootstrapping algorithm, otherwise under default parameters. Nodes of the ML tree with BSP≥70 were significantly supported.

Uncorrected pairwise distance (*p*-distances) of the sequenced cyt *b* data among congeners was calculated using MEGA 6 (Tamura et al., 2013).

### Morphological analyses

In addition to the three newly collected specimens, a total of 33 vouchered specimens were also examined for morphological data (Appendix). As the genus is understudied and contains cryptic diversity, only topotypic or paratopotypic specimens were examined if available to avoid taxonomic confusion. Additional museum abbreviations include Yibin University, Yibin, China (YBU); Museum of Biology, Sun Yat-Sen University, Guangzhou, China (SYS); Institute of Ecology and Biological Resources, Vietnamese Academy of Science and Technology, Hanoi, Vietnam (IEBR); Zoological Museum, Vietnam National University, Hanoi, Vietnam (VNUH).

Measurements were taken by Jin-Long Ren with a digital slide-caliper to the nearest 0.1 mm, except for total lengths (TL), which were measured using a measuring tape to the nearest 1 mm. Measurement methods and their definitions followed Zhao (2006), and included: total length (TL), snout-vent length (SVL), tail length (TaL), head length (HL), and head width (HW). In addition, the following morphometric characters were examined in this study: rostral length (RL): distance from tip of snout to anterior edges of eyes; rostral width (RW): maximum distance between supralabials at the anterior edges of eyes; interorbital distance (IOD): distance between upper edges of eyes; eye width (EW): maximum horizontal eye width; distance between the lower margins of eye and of lip (SoL); maximum loreal length (LoL); maximum loreal depth (LoD); maximum anterior temporal length (ToL); and maximum anterior temporal depth (ToD).

The following pholidosis characteristics were also recorded (character definition and counting methods followed Zhao (2006)): internasal counts (IN), prefrontal counts (PrF), frontal counts (F), parietal counts (P), loreal counts (L), preocular counts (PrO), postocular counts (PtO), supraocular counts (SpO), subocular counts (SbO), supralabial counts (SL), infralabial counts (IL), temporal counts (TEM), chin shield counts, maxillary tooth counts (MT), dorsal scale row counts (DSR), ventral counts (VEN), and subcaudal counts (SC). Dorsal scale rows (DSR) were taken at one head length (HL) behind head, at midbody, and at one head length (HL) before cloaca, respectively. Symmetric characters (e.g., temporals and supralabials) were given in left/right order and averages were used in the analyses, except for maxillary teeth, which were counted on the left side of each specimen only.

In addition to the vouchered specimens examined (Appendix), morphological data of congeners were also obtained from the literature, including original descriptions and redescriptions (Angel & Bourret, 1933; Boulenger, 1888, 1899, 1903, 1914; Cope, 1895; David et al., 2011, 2015; Günther, 1872; Li et al., 2010; Pope, 1928; Stuart & Chuaynkern, 2007; Teynié et al., 2014; Wang et al., 2017; Yang et al., 2013; Zhao et al., 1978, 1998; Zhao, 1999, 2005, 2006; Ziegler et al., 2008).

## RESULTS

For the 29 aligned sequences of the congeners, a total of 406 variable and 367 parsimony informative sites were identified. The uncorrected pairwise sequence divergence of 11 sampled *Opisthotropis* species ranged from 5.0% to 17.8% (Table 2). The three sampled individuals of the Hunan population shared identical haplotypes for cyt *b* and possessed a genetic divergence of 5.6% and 5.1% from *O*. *cheni* and *O*. *latouchii*, respectively. Such divergences are higher than the recognized species level in *Opisthotropis* (5.0% between *O*. *cheni* and *O*. *latouchii*), with the intraspecific genetic divergences of *O*. *latouchii* and *O*. *cheni* ranging from 0.0% to 0.5%.

**Table 2:** Uncorrected pairwise sequence divergence among cyt *b* mtDNA gene sequences of *Opisthotropis* species

	A	B	C	D	E	F	G	H	I	J	K	L	M	N	O
*O*. *zhaoermii* **sp. nov.** (MG012799) (A)															
*O*. *zhaoermii* **sp. nov.** (MG012800) (B)	0.0%														
*O*. *zhaoermii* **sp. nov.** (MG012801) (C)	0.0%	0.0%													
*O*. *cheni* (GQ281779) (D)	5.6%	5.6%	5.6%												
*O*. *cheni* (KY594741) (E)	5.6%	5.6%	5.6%	0.5%											
*O*. *latouchii* (GQ281783) (F)	5.1%	5.1%	5.1%	5.1%	5.0%										
*O*. *latouchii* (KY594742) (G)	5.1%	5.1%	5.1%	5.1%	5.0%	0.0%									
*O*. *kuatunensis* (KY594745) (H)	14.8%	14.8%	14.8%	14.2%	14.3%	13.8%	13.8%								
*O*. *lateralis* (GQ281782) (I)	16.1%	16.1%	16.1%	15.6%	15.6%	15.9%	15.9%	16.5%							
*O*. *laui* (KY594738) (J)	16.4%	16.4%	16.4%	16.5%	16.6%	16.3%	16.3%	16.8%	15.0%						
*O*. *maxwelli* (KY594736) (K)	15.2%	15.2%	15.2%	15.9%	15.8%	15.5%	15.5%	15.9%	13.3%	11.4%					
*O*. *andersonii* (KY594732) (L)	16.0%	16.0%	16.0%	17.4%	17.3%	16.0%	16.0%	16.7%	14.8%	12.2%	11.8%				
*O*. *shenzhenensis* (KY594727) (M)	16.2%	16.2%	16.2%	17.2%	17.3%	16.5%	16.5%	17.8%	14.8%	14.3%	11.2%	11.7%			
*O*. *maculosa* (KY594748) (N)	16.7%	16.7%	16.7%	17.5%	17.4%	16.5%	16.5%	15.9%	17.7%	16.5%	15.0%	16.7%	16.5%		
*O*. *guangxiensis* (GQ281776) (O)	15.9%	15.9%	15.9%	16.6%	16.3%	15.5%	15.5%	15.7%	16.9%	16.3%	15.5%	14.9%	16.6%	16.0%	

**Figure 5:**
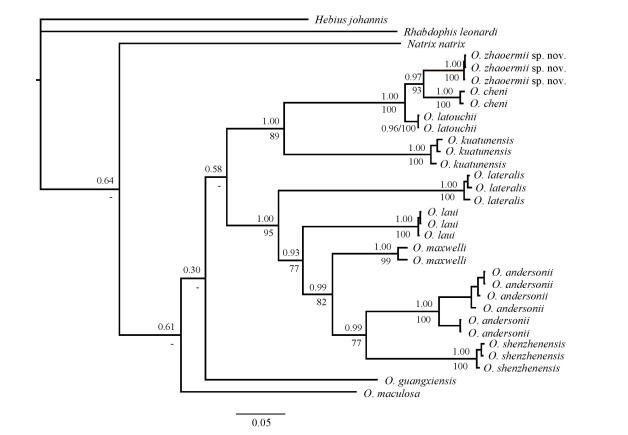
BI phylogenetic tree estimated from cyt *b* sequences depicting phylogenetic relationships of *Opisthotropis* (numbers above branches are BPP and numbers below branches are BSP ( > 70 retained))

For phylogenetic analyses, a majority rule consensus tree inferred from BI (-ln *L*=-6 531.011 3; average standard deviation of split support=0.002 005; ESS > 200) was consistent with the ML tree (Figure 5). *Opisthotropis* was recovered as monophyletic with current samplings. Although interspecific relationships among congeners within *Opisthotropis* were not fully resolved, the final consensus tree yielded high support (BPP > 0.95; BSP > 70) for key nodes concerning the relationship between the Hunan population with recognized congeners. The Hunan population was recovered as a monophyletic clade (BPP=1.00; BSP=100) within *Opisthotropis*, which was most closely related to *O*. *cheni* (BPP=0.97; BSP=93). *Opisthotropis* *latouchii* is the sister taxon to the node, and together all three species constitute the *O*. *latouchii* species group (Figure 5).

Detailed morphological comparisons showed that the Hunan population possessed a suite of morphological characteristics that can be readily distinguished from all recognized congeners, including number of maxillary teeth, head scalation, and ornamentation patterns (Table 3; Figures 1–4). Therefore, according to both molecular and morphological evidence, we describe the Hunan population of *Opisthotropis* as a new species below.

**Table 3:** Morphological characteristics of the examined specimens of *O*. *zhaoermii* sp. nov., *O*. *cheni*, and *O*. *latouchii* examined in this study

	*O*. *zhaoermii* **sp. nov.**		*O. latouchii*		*O. cheni*	
	male (*n*=1)	female (*n*=2)	male (*n*=7)	female (*n*=3)	male (*n*=5)	female (*n*=4)
TL (mm)	529.7	514.8-586.7	384.4-463.6	401.2–446.3	395.9–564.5	433.0–534.7
TaL (mm)	108.62	101.26–120.49	78.89–92.86	82.59–91.25	88.52–113.48	86.45–108.21
Tal/TL	0.21	0.20–0.21	0.20–0.21	0.20–0.21	0.20–0.22	0.18–0.21
HL (mm)	12.72	12.92–13.61	9.94–11.69	9.43–11.13	10.40–14.03	12.33–13.64
HW (mm)	8.76	9.81–10.84	7.11–8.34	7.26–8.53	8.55–10.25	8.22–9.80
HL/HW	1.45	1.26–1.32	1.31–1.46	1.25–1.30	1.21–1.53	1.32–1.50
RL (mm)	4.60	4.57–4.78	3.18–4.29	3.18–3.55	3.59–4.56	4.21–4.58
RW (mm)	7.14	7.00–8.20	4.87–6.27	5.62–6.66	6.46–7.44	6.95–7.14
RL/RW	0.64	0.56–0.68	0.52–0.70	0.53–0.58	0.56–0.67	0.61–0.65
IOD (mm)	5.41	5.72–6.10	4.20–5.47	4.65–4.82	4.65–5.68	4.94–6.05
IOD/HW	0.62	0.56–0.58	0.54–0.66	0.57–0.64	0.54–0.64	0.54–0.62
EW (mm)	1.33	1.36–1.60	0.87–1.09	0.96–1.08	1.37–1.51	1.29–1.48
EW/HL	0.10	0.11–0.12	0.08–0.10	0.09–0.11	0.10–0.13	0.10–0.12
SOL (mm)	1.94	1.94–1.98	1.32–1.64	1.26–1.44	1.50–1.94	1.46–1.85
SOL/HL	0.15	0.15	0.13–0.16	0.13–0.14	0.13–0.15	0.12–0.14
LoL (mm)	2.10	2.20–2.32	1.47–1.85	1.44–1.49	1.78–2.46	2.11–2.42
LoD (mm)	1.31	1.04–1.28	0.84–1.12	0.69–0.86	1.02–1.39	1.13–1.30
LoL/LoD	1.64	1.82–2.11	1.56–1.96	1.68–2.18	1.75–1.89	1.73–2.07
ToL (mm)	2.06	2.84–3.01	2.00–2.66	1.99–2.46	2.48–3.56	2.57–3.42
ToD (mm)	1.19	1.41–1.47	0.85–1.09	0.94–1.00	0.95–1.09	0.75–1.19
ToL/ToD	1.74	2.02–2.04	1.87–2.80	2.08–2.66	2.36–3.63	2.88–3.47
DSR	17:17:17	17:17:17	17:17:17	17:17:17	17:17:17	17:17:17
SL	9	8–9	8–9	7–9	7–9	8–9
IL	8	8–10	7–9	8–9	8–10	9–10
TEM	1+2+2/1+1+2	1+2/1	1+1/2	1+1/2	1+2/1	1+1
MT	29	28–30	18–24	12–23	25–28	25–28
VEN	152	147–152	155–161	155–159	152–162	146–153
SC	62	54–61	57–63	59–63	61–64	47–60
Abbreviations are listed in Material and Methods.						

### Taxonomic account

***Opisthotropis zhaoermii* sp. nov., Ren, Wang, Jiang, Guo, Li, 2017** (Figures 1, 2, 3A, 4A)

**Holotype** CIB109999 (field No. HNYS 20170049) (Figures 1A, 1B, 2A, 2B, 3A, 4A), adult female, from Zuolong Gorges, Guzhang, Tujia-Miao of western Hunan, China (N28°42'17.88" , E109°55'26.26" , 561 m a.s.l., Figure 6), collected by Jin-Long Ren and Si-Bo Su on 24 August 2017.

**Paratypes** One adult male, CIB109998 (field No. HNYS20170019) (Figures 1C, 1D, 2C, 2D) and one adult female CIB110000 (field No. HNYS20170050), collected by Jin-Long Ren and Si-Bo Su at the same locality as holotype on 22 and 23 August 2017.

**Diagnosis** A large-sized species of *Opisthotropis* diagnosed by the following morphological characters: (1) head barely distinct from neck; (2) body and tail moderately slender; (3) prefrontal single, much broader than long; (4) nostrils directed upwards; (5) eyes small; (6) maxillary teeth subequal; (7) anterior neck dorsal scales smooth, middle body with faint keels, tending to moderately keeled rear body and on tail.

The new species differs from all congeners by a combination of the following characters: (1) body size large (TL 514–586 mm); (2) tail moderate (Tal/TL 0.20–0.21); (3) dorsal scale rows 17:17:17; (4) ventral scales 147–152; (5) subcaudal scales 54–62; (6) preocular absent; (7) loreal elongated and entering orbit; (8) supralabials mostly 9 (rarely 8), fifth and sixth entering obit; (9) anterior temporals short, ToL/ToD 1.74–2.04; (10) maxillary teeth 28–30; (11) irregular yellow stripes edged with ochre present on dorsal and lateral head; (12) clear black and yellow longitudinal streaks present on dorsal and lateral body, some streaks fused to light patches or stripes anteriorly; (13) venter pale yellow in life, with asymmetric blackish speckles along outer margins.

**Description of holotype** Body relatively large (TL 586 mm), stout and cylindrical; head short and broad, HL/SVL 0.03, HL/HW 1.26, dorsally depressed, not distinct from neck; eyes small, EW 1.60 mm, EW/HL 0.12; pupils round; interorbital distance medium, IOD/HW 0.56. Nostrils oval, directed upwards, located at inner side of nasals; tail long, TaL/TL 0.21, tapering posteriorly. Maxillary teeth 30, subequal, densely set, not grooved.

Rostral semi-circular, width 1.5 times longer than high, visible from above; nasals subhexagonal, in contact with rostral, not divided, furrow from inferior nostril to edge of nasal, reaching superior margin of second supralabial; internasals quadrangular, longer than wide, away from loreal, contacting nasals laterally, prefrontal posteriorly; prefrontal single, subrectangular, twice as broad than long, protruding anteriorly, posterior edge broadly in contact with frontal, lower corner in contact with supraoculars on both sides; frontal single, subpentagonal, somewhat peach-shaped, slightly broader than long, 1.8 times longer than prefrontal; single pair of parietals present, 1.5 times longer than wide; loreals 1/1, elongated, subrectangular in shape, twice as long than high, about 1.4 times longer than eye diameter, entering orbit, in contact with nasal, prefrontal, supraocular, supralabials (third, fourth, fifth). Preocular absent; postoculars 2/2, upper pair slightly shorter than eye, larger than lower pair; lower postocular in contact with sixth supralabial on left, sixth and seventh on right; supraocular 1/1, large, "B"-shaped, about equal length to loreal; supralabials 8/9, fifth and sixth entering orbit. On left, supralabials 1–5 distinctly higher than long, supralabials 6–8 longer than high; on right, supralabials 1–6 distinctly higher than long, equal size in seventh, last two longer than high. Temporals 1+2/1+2, anterior ones largest, about two times longer than wide, in contact with supralabials 6–7 on left side, supralabials 7–9 on right; two posterior temporals shorter and smaller than anterior pair, subequal, slightly shorter than loreals; two pairs of chin shields present, anterior ones about 1.2 times longer than posterior ones; posterior chin shields separated from each other by two scales; mental groove apparent; infralabials 9/8, first four in contact with anterior chin shields, fourth and fifth in contact with posterior chin shields; sparse minute granular asperities on head scales. Dorsal scale rows 17:17:17 without apical pits, vertebral not enlarged, dorsal scales smooth anteriorly, gradually keeled from seventh ventral position, weakly keeled anteriorly and stronger posteriorly; outer most dorsal scale rows along both sides of body smooth entirely before cloaca. Ventrals 152, precloacal divided; anal divided; subcaudal 60, paired, with single terminal rod.

**Coloration of holotype in life:** In life, the dorsal surface of the head is brownish yellow, mottled with small brownish black spots and patches. Lighter irregular markings are present on the internasals, prefrontal, and frontal, which do not sharply separate the darker center from the brownish yellow background color. Brownish ocher stripes are observed on the posterior edges of frontal and parietals, forming several irregular broken boundary lines. In front of the stripes on the parietals, six thicker oblique stripes are observed. Color patterns are similar on the lateral surface, supralabials 3–6 (3–7 on right) and infralabials 2–8 brownish yellow with anterior blackish borders. The blackish edge of upper part of supralabials 6–8 (7–9 on right), postoculars, and lower part of anterior temporal forming a thick "eyebrow" streak pattern, connecting with the outer most irregular black lateral stripe extended to tail tip. From supralabial 5 to neck, darker brownish yellow background color gradually transitions into light yellow. Tongue is purple with pink markings, gradually turning white at apex. Dorsal scales from row 3 to 15, each scale is black with a subtriangular or rectangular yellow center, thus constituting thirteen clear longitudinal stripes along the whole trunk just after the posterior edges of the parietals. Before 24th ventral position, dorsal scale rows 1 and 17 pale yellow entirely or marked with minute black spots, black upper edges gradually appearing on these two rows of scales after 24th ventral position, forming an extra yellow stripe along each side. Along the vertebral scales on the back, these yellow stripes fuse to seven conspicuous irregular patches or stripes anteriorly, only eight yellow stripes observed at the base of the tail. Chin and anterior venter pale yellow, venter becoming lighter posteriorly. The ventral scales are cream with bold yellow posterior margins, asymmetric black spots present along the outer margins, somewhat transparent. Subcaudals resemble ventrals but with a black longitudinal joining stripe after the eleventh subcaudal scale.

**Coloration in preservation** Specimens fixed in formalin and preserved in ethanol resemble the coloration of live animals. However, the yellow longitudinal stripes faded to a dull yellow hue, and the cream venter changed to uniform beige and was no longer transparent (Figures 2A, 2B).

**Variation** Paratypes generally resemble holotype in morphological characters (Figures 1C, 1D, 2C, 2D; Table 3). However, the male paratype CIB109998 differs from the female specimens (CIB109999 and CIB 110000) by smaller body size (TL 514 mm vs. 529–586 mm in females), shorter anterior temporals (ToL/ToD 1.74 vs. 2.02–2.04 in females), and rather irregular yellow patterns within each dorsal scale (vs. regular) (Figures 1C, 1D, 2C, 2D). As the sample size is small, it is unknown whether such differences observed between the two sexes represent sexual dimorphism in this species.

**Figure 7:**
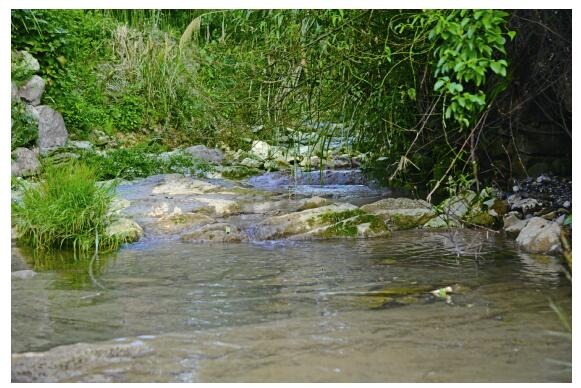
Travertine stream habitat of *Opisthotropis zhaoermii* sp. nov. in western Hunan, China (Photo by Jin-Long Ren)

**Distribution and natural history** This species is only known from the type locality presently (Figure 6). *Opisthotropis zhaoermii* **sp. nov.** inhabits small, fast-flowing mountain streams in forested areas, with water temperature and pH between 19.9–21.2 ℃ and 7.85–7.93, respectively. Being nocturnal, individuals were seen swimming at the edge of the backwater of travertine waterfalls from 2100h to 0100h at night (Figure 7). The holotype was collected during a heavy rainstorm that caused the water to become extreme turbid, with water temperature and pH measurements of 19.5 ℃ and 8.13, respectively.

When handled, individuals never stroked or displayed threating postures. Instead, individuals struggled violently and released a musky, pungent, and enduring defensive odor, much like that of *O*. *balteata* and *O*. *kuatunensis* (Pope, 1935). The scales of the snakes become dehydrated and crimped quickly after leaving the water for about 10 min, but recovered rapidly when returned to water. Similar to other congeners in China, the new species may prey on earthworms, tadpoles, freshwater isopods, crabs, and small fish (Pope, 1935). Other herpetofauna, including *Quasipaa boulengeri*, *Odorrana schmackeri*, *Lycodon rufozonatum*, *L*. *fasciatus*, and *Deinagkistrodon acutus*, were observed sympatric with the new species.

**Etymology** The specific epithet, *zhaoermii*, is derived from the name of an internationally renowned Chinese herpetologist, Prof. Er-Mi Zhao, who unfortunately passed away on 24 December 2016. Designation of this specific epithet honors his great contribution to herpetological research in China, specifically to his contribution to the taxonomy of *Opisthotropis*, including the first description of the sister species (i.e., *O*. *cheni*, see above) of the newly found species. We suggest Zhao's Mountain Stream Snake as its English common name, and "Zhao Shi Hou Leng She" (赵氏后棱蛇) as its Chinese common name.

**Comparisons** For pholidosis characteristics, *Opisthotropis* *zhaoermii* **sp. nov.** is most similar to *O*. *cheni* and *O*. *latouchii* in having the same number of dorsal scale rows (17:17:17) and postoculars (2), and all three species possessing elongated loreals that enter orbits. However, *Opisthotropis zhaoermii* **sp. nov.** can be distinguished from both recognized species by a suite of morphological characteristics. The new species differs from *O*. *cheni* by having a higher number of maxillary teeth (28–30 vs. 25–28), a different temporal format (1+2 [rarely 1 only] vs. 1+1, occasionally 1+2), shorter anterior temporals (ToL/ToD 1.74–2.04 vs. 2.36–3.63), a larger distance between the lower margins of the eye and of lip (SoL/HL 0.15 vs. 0.12–0.14), and distinct dorsal color patterns (alternate, longitudinal black and yellow stripes vs. uniform dark olive with light-yellow transverse bands); from *O*. *latouchii* by having a larger body size (TL 514–586 mm vs. 360–419 mm), a higher number of maxillary teeth (28–30 vs. 12–25), lower ventral counts (147–152 vs. 155–159), a different temporal format (1+2 [rarely 1 only] vs. 1+1, occasionally 1+2), and a distinct dorsal color pattern (alternate longitudinal stripes partly fused vs. distinct stripes never fused together) (Figures 3, 4; Table 3).

For other congeners that also have 17:17:17 dorsal scale rows, *Opisthotropis* *zhaoermii* **sp. nov.** differs from *O*. *lateralis* and *O.* *maxwelli* by having a different loreal position (loreals entering orbits vs. separate from orbits). In addition, the new species differs from *O*. *lateralis* by having a lower number of supralabials (8–9 vs. 10–11) and a larger size (TL 514–586 mm vs. 294–461 mm); from *O*. *maxwelli* by having a higher number of maxillary teeth (28–30 vs. 22–23) and a higher number of supralabials (8–9 vs. 6–7). Furthermore, *Opisthotropis* *zhaoermii* **sp. nov.** differs from *O*. *andersonii* by having a higher number of maxillary teeth (28–30 vs. 22–23), a larger body size (TL 514–586 mm vs. 240–462 mm), and a longer tail (TaL/TL 0.20–0.21 vs. 0.15–0.20); from *O*.*daovantieni* Orlov, Darevsky and Murphy, 1998 and *O*. *spenceri* Smith, 1918 by having a different internasals location (separated from loreals vs. in contact) and fewer ventrals (147–152 vs. 189–194 in *O*. *daovantieni* and 178–185 in *O*. *spenceri*).

For congeners that have 17 rows of dorsal scales at the midbody, including *O*. *rugosa* (Lidth de Jeude, 1890), *O*. *tamdaoensis* Ziegler, David and Vu, 2008, and *O*. *atra* Günther, 1872, the new species differs from *O*. *rugosa* and *O*. *tamdaoensis* by having a different dorsal scale formula (17:17:17 vs. 19:17:15 in *O*. *rugosa*, 19:17:17 in *O*. *tamdaoensis*), and from *O*. *atra* by lower number of ventrals (147–152 vs. 170), lower subcaudal counts (54–62 vs. 65), and higher number of supralabials (8–9 vs. 7).

For the remaining congeners, *Opisthotropis* *zhaoermii* **sp. nov.** differs from *O. guangxiensis*, *O*. *jacobi*, *O*. *kikuzatoi* Okada and Takara, 1958, and *O*. *maculosa* by having different dorsal scale rows at the midbody (17 vs. 15) and a lower number of ventrals (147–152 vs. 164–174 in *O. guangxiensis*, 155–179 in *O*. *jacobi*, 180–183 in *O*. *kikuzatoi*, and 166–188 in *O*. *maculosa*); from *O*. *balteata*, *O*. *kuatunensis*, *O*. *shenzhenensis*, *O*. *durandi* Teynié, Lottier, David, Nguyen and Vogel, 2013, and *O*. *laui*, 2013 by a distinct formula of dorsal scale rows (17:17:17 vs. 19:19:17 in *O*. *balteata*, 19:19:19 in *O*. *kuatunensis* and *O*. *shenzhenensis*, 19:21:17 in *O*. *durandi*, and 25:23:23 in *O*. *laui*); from *O*. *cucae* David, Pham, Nguyen and Ziegler, 2011 by having a distinct position of internasals (truncated anteriorly and separate from loreal vs. curved and in contact with loreal); from *O*. *typica* (Mocquard, 1890) and *O*. *alcalai* Brown and Leviton, 1961 by having a distinct prefrontal condition (complete vs. divided).

### Key to species of *Opisthotropis*

With the description of this new species, an updated diagnostic key to all species of the genus *Opisthotropis* is provided here to facilitate future taxonomic work.

1  Prefrontal single................................................................. 2

–  Prefrontal divided............................................................. 21

2  Dorsal scale rows 15 at midbody...................................... 3

–  Dorsal scale rows 17–23 at midbody................................ 6

3  Supralabials 6–7; dorsal scales keeled posteriorly............................................*O*. *kikuzatoi*

–  Supralabials 7–10; dorsal scales smooth entirely........... 4

4  Dorsal scale rows 17:15:15; dorsal surface with pale yellow crossbars........................................*O*. *guangxiensis*

–  Dorsal scale rows 15:15:15; dorsal surface without pale yellow crossbars..............................5

5  Anterior chin shields nearly two times longer than posterior ones; dorsal surface uniformly blackish brown..........*O.* *jacobi*

–  Anterior chin shields and posterior ones about equal length; dorsal surface dark with a yellow spot on each dorsal scale........................................*O*. *maculosa*

6  Dorsal scale rows 17 at midbody...................................... 7

–  Dorsal scale rows 19–23 at midbody.............................. 16

7  Ventral scales 189–194................................. *O*. *daovantieni*

–  Ventral scales less than 189.............................................. 8

8  Anterior temporal scales 2; subocular present............................*O*. *tamdaoensis*

–  Anterior temporal scales 1; subocular absent.................. 9

9  Dark lateral stripe abruptly separating dark dorsum from light ventral color; loreal not entering orbit........................................*O*. *lateralis*

–  No such lateral stripe, loreal entering orbit or not........ 10

10  Loreal not entering orbit, LoL/LoD 1.40–1.70..........*O*. *maxwelli*

–  Loreal entering orbit or not, LoL/LoD > 1.70.................... 11

11  Tail short, TaL/TL 0.15–0.20........................... *O*. *andersonii*

–  Tail moderate or long, TaL/TL≥0.20................................ 12

12  Preocular absent; loreal elongated and entering orbit..........13

–  Preocular present; loreal normal-sized and not entering orbit........................................15

13  Body size small, total length shorter than 419 mm; maxillary teeth count no more than 25..........*O*. *latouchii*

–  Body size moderate or large, total length longer than 419 mm; maxillary teeth count no less than 25....................14

14  Maxillary teeth count 25–28; anterior temporal scales elongated, ToL/ToD 2.63–3.63; dorsal surface dark olive with yellow crossbars..............................*O*. *cheni*

–  Maxillary teeth count 28–30; anterior temporal scales short, ToL/ToD 1.74–2.04; dorsal surface with longitudinal yellow stripes....................*O*. *zhaoermii* **sp. nov.**

15  Nasal scales paired on both sides.......................... *O*. *atra*

–  Nasal scales not paired on both sides........... *O*. *spenceri*

16  Dorsal scale rows 19 at midbody.................................... 17

–  Dorsal scale rows 21 or 23 at midbody.......................... 20

17  Internasal in contact with loreal........................... *O*. *cucae*

–  Internasal not in contact with loreal............................... 18

18  Dorsal scale rows 19:19:17; ventral scales 190–205...................*O*. *balteata*

–  Dorsal scale rows 19:19:19; ventral scales less than 190........................................19

19  Supralabials 9–10, not divided horizontally; dorsal scales feebly keeled anteriorly, strongly keeled posteriorly; dorsal surface olive-green with fine black mesh pattern........................................................*O*. *shenzhenensis*

–  Supralabials 13–16, divided horizontally; dorsal scales strongly keeled entire body; dorsal surface dark brown with black longitudinal lines....................................................................... *O*. *kuatunensis*

20  Dorsal scale rows 21 at midbody....................... *O*. *durandi*

–  Dorsal scale rows 23 at midbody............................. *O*. *laui*

21  Dorsal scale smooth............................................. *O*. *alcalai*

–  Dorsal scale keeled.......................................................... 22

22  Dorsal scale rows 17 at midbody; preocular 1; supralabials 10–11....................*O*. *rugosa*

–  Dorsal scale rows 19 at midbody; preocular 2; supralabials 11..............................*O*. *typica*

## DISCUSSION

Although recent taxonomic work has shed light on the taxonomy of Chinese *Opisthotropis*, research has been focused on the Pearl River Valley only (Wang et al., 2017; Yang et al., 2013), and diversity of the genus in most parts of southern China remained overlooked. Our discovery of the new species *Opisthotropis zhaoermii* in the Yangtze River Basin highlights the underestimated diversity of the genus in southern China and calls for further fieldwork and continuous taxonomic studies of the group in the under-surveyed regions of southern China.

Despite the recent taxonomic discoveries of *Opisthotropis* in southern China and northern Indochina, many of the newly described species are known from only a handful of specimens, and in some case, from a single holotype from the type locality (David et al., 2011; Yang et al., 2013; Ziegler et al., 2008). The low numbers of vouchered specimens and geographic samplings have resulted in limited knowledge on the morphological variations, distribution patterns, and conservation of members of the genus. Furthermore, few studies have reported detailed natural history data of congeners, including color in life, behaviors, reproductive biology, and population statuses. As ecological niche differentiation may be a driver of species diversification in *Opisthotropis* (Yang et al., 2011), it is important to collect ecological data for different congeners. Here we reported on water temperature and pH data of an *Opisthotropis* species for the first time, and recommend that future studies collect ecological data of *Opisthotropis* in the field, including the addition of more parameters such as flow speed of water, canopy coverage, humidity, and prey community structures. We recommend future studies to explore the ecology of this unique group of aquatic snakes and investigate the possible ecological speciation mechanisms of the group.

## ACKNOWLEDGEMENTS

We thank Mr. Si-Bo Su (CIB), Mr. Hai-Yun Peng, Mr. Dan Xiang, and Mr. Tao Xiang for their help in the field; Ms. Tong Yang (CIB) and Ms. Guan-Nan Wen (CIB) for their assistance in the laboratory; Mr. Han-Ren Liu, Mr. Yu-Lin Xie (YBU), Mr. Ping Wang (YBU), and Ms. Xin-Hong Xie (YBU) for their kind help in morphological data collection; and Professor Yue-Zhao Wang (CIB), Professor Yue-Ying Chen (CIB), and Mr. Ke Lu (CIB) for the loan and examination of specimens.

## APPENDIX

The following specimens were examined in this study:

*opisthotropis cheni* (*n*=9): cib98273, ybu12110, ybu071040, 071042, 071046–071050, nanling national nature reserve, ruyuan county, guangdong province, china.

*Opisthotropis cucae* (*n*=1): IEBR A.0924 (holotype), Chu Mom Ray National Park, Sa Thay District, Kon Tum Province.

*Opisthotropis latouchii* (*n*=10): CIB9983, 9988, 9990–9994, 9998–10000, Tongmu Village, Fujian Province, China.

*Opisthotropis lateralis* (*n*=12): YBU14465, 14477–14478, Fulong Township, Fangchenggang City, Guangxi Province, China; 14516–14523, Mt Pingtian, Guigang City, Guangxi Province, China; 091014, Guangxi Province, China.

*Opisthotropis tamdaoensis* (*n*=1): VNUH010606 (holotype), Tam Dao, Vinh Phuc Province, Vietnam.

*Opisthotropis zhaoermii* **sp. nov.** (*n*=3): CIB109998 (paratype), CIB109999 (holotype), CIB110000 (paratype), Zuolong Gorges, Guzhang County, Tujia-Miao Autonomous Prefecture, Hunan Province, China.
